# The Cell-Free Expression of MiR200 Family Members Correlates with Estrogen Sensitivity in Human Epithelial Ovarian Cells

**DOI:** 10.3390/ijms21249725

**Published:** 2020-12-20

**Authors:** Éva Márton, Alexandra Varga, Lajos Széles, Lóránd Göczi, András Penyige, Bálint Nagy, Melinda Szilágyi

**Affiliations:** 1 Department of Human Genetics, Faculty of Medicine, University of Debrecen, H-4032 Debrecen, Hungary; marton.eva@med.unideb.hu (É.M.); varga.alexandra@med.unideb.hu (A.V.); szelesl@med.unideb.hu (L.S.); goczi.lorand@med.unideb.hu (L.G.); penyige@med.unideb.hu (A.P.); nagy.balint@med.unideb.hu (B.N.); 2 Faculty of Pharmacology, University of Debrecen, H-4032 Debrecen, Hungary

**Keywords:** ovarian cancer, estrogen, zeralenone, bisphenol A, estrogen receptor, cell-free miRNA, miR200, miR203a, EMT

## Abstract

Exposure to physiological estrogens or xenoestrogens (e.g., zearalenone or bisphenol A) increases the risk for cancer. However, little information is available on their significance in ovarian cancer. We present a comprehensive study on the effect of estradiol, zearalenone and bisphenol A on the phenotype, mRNA, intracellular and cell-free miRNA expression of human epithelial ovarian cell lines. Estrogens induced a comparable effect on the rate of cell proliferation and migration as well as on the expression of estrogen-responsive genes (*GREB1*, *CA12*, *DEPTOR*, *RBBP8*) in the estrogen receptor α (ERα)-expressing PEO1 cell line, which was not observable in the absence of this receptor (in A2780 cells). The basal intracellular and cell-free expression of miR200s and miR203a was higher in PEO1, which was accompanied with low *ZEB1* and high E-cadherin expression. These miRNAs showed a rapid but intermittent upregulation in response to estrogens that was diminished by an ERα-specific antagonist. The role of ERα in the regulation of the *MIR200B-MIR200A-MIR429* locus was further supported by publicly available ChIP-seq data. MiRNA expression of cell lysates correlated well with cell-free miRNA expression. We conclude that cell-free miR200s might be promising biomarkers to assess estrogen sensitivity of ovarian cells.

## 1. Introduction

Cancer is the second leading cause of death worldwide. Among women, ovarian cancer is considered to be the fifth most common cause of cancer death and it is the most lethal form of gynecological malignancy [1]. It is generally accepted that high estrogen exposure increases the risk for gynecological cancers including ovarian cancer. This represents a great concern among postmenopausal women because estrogens are frequently used to prevent age-related diseases including osteoporosis or stroke [2,3,4,5]. Accumulating evidence suggests that exposure to xenoestrogens also affects cancer rates. Xenoestrogens can be defined as chemicals that mimic the effect of physiological estrogens. They can be divided into natural compounds (e.g., produced by fungi, such as mycoestrogens e.g., zeralaenone, ZEA) and synthetic agents (e.g., bisphenol A, BPA). ZEA is a mycotoxin produced by *Fusarium* sp., which is one of the most economically important fungal genera. They frequently infest crops in the field or during storage and thus ZEA is one of the most frequently isolated mycotoxins in cereals, mainly in maize [6,7,8]. BPA is a carbon-based synthetic compound that is used to make certain plastics and epoxy resins and is one of the highest volume chemicals produced worldwide [9,10]. BPA tends to leach from plastic items, thus human contamination is considered to be high especially in industrialized countries, which is supported by the observation that BPA has been detected in human serum, urine, amniotic fluid and breast milk [9,10]. Both ZEA and BPA are confirmed to have an estrogen-disruptive effect in domestic animals and humans and their toxicity is also suggested by several studies [9,10,11,12,13].

Estrogens contribute to the development of cancer by affecting the expression of several genes involved in cellular proliferation, apoptosis or DNA repair. Furthermore, estrogens support metastasis formation through promoting tumor cell migration by inducing epithelial-to-mesenchymal transition (EMT), where tumor cells lose their epithelial features and gain a mesenchymal phenotype [2,14]. Estrogen signaling is mostly mediated by estrogen receptors (ER) that consist of two subtypes: ERα and ERβ. Both of them are members of the nuclear receptor superfamily and function as transcription factors in response to ligand attachment [15]. ERα-mediated estrogen signaling was shown to be crucial in the development of ovarian cancer, supported by the observation that ERα is frequently overexpressed in ovarian tumors, and that anti-estrogen treatments such as the application of tamoxifen or aromatase inhibitors improves survival in certain subtypes of ovarian cancer [2,16,17]. On the other hand, ERβ is considered to have an antiproliferative effect and shows overexpression in normal tissues [16,17,18]. It is important to mention that both BPA and ZEA are able to interact with ERα according to previous studies [9,19]. Estrogen action is also mediated through ER-independent pathways (e.g., by G-protein coupled ERs) and the significance of epigenetic regulatory mechanisms has also been confirmed recently [15]. Furthermore, accumulating evidence suggests that miRNAs also have a pivotal role in estrogen response. Several miRNAs have been shown to be upregulated or downregulated after exposure to estrogens and ERα expression proved to be directly regulated by miRNAs [20].

Although the carcinogenic effect of estrogens is a well-known phenomenon in breast cancer [3], limited information is available on their significance in the development of ovarian cancer, especially in the respect of cell-free miRNAs. Cell-free miRNAs are small non-coding RNA molecules present in the extracellular fluid (including body fluids) that are considered to play a prominent role in cell-to-cell communication and are promising biomarkers in the non-invasive diagnosis of several diseases [21,22,23]. In our previous studies we identified several cell-free miRNAs that are over-represented in the plasma samples of patients with ovarian carcinoma compared to healthy controls [24,25]. In this study our goal was to monitor the effect of estradiol (E2), ZEA and BPA on miRNA expression regarding both intracellular and cell-free miRNAs. We present here that E2, ZEA and BPA induce comparable changes in the phenotype as well as in the expression of estrogen responsive genes in human epithelial ovarian cells. We also show that estrogens and ERα affect the expression of intracellular and cell-free counterparts of miR200 family members and miR203a, which have an influence on E-cadherin expression and the migratory ability of PEO1 and A2780 cells.

## 2. Results

### 2.1. Estradiol, Zearalenone and Bisphenol A Induce Comparable Phenotypic Changes in Ovarian Cells

Two human epithelial ovarian cell lines were used in the study. PEO1 is considered to be an estrogen-responsive cell line that highly expresses ERα [26,27]. ERα expression of PEO1 was confirmed by qPCR prior to our studies (relative expression: 0.3 ± 0.1 relative to *GAPDH*). The expression of ERα was not detectable by qPCR in the A2780 cell line in our measurements, which is in good agreement with previous results [18,26]. Therefore this cell line was used as a negative control in our study. First the estrogen response induced by ZEA and BPA was compared to the effect of E2. The influence of E2, ZEA and BPA molecules on growth rate, migration, apoptosis and cell lysis was assessed. Cells were treated with various concentrations (1–1000 nM) of the agonists in order to compare their effective doses.

**Figure 1:**
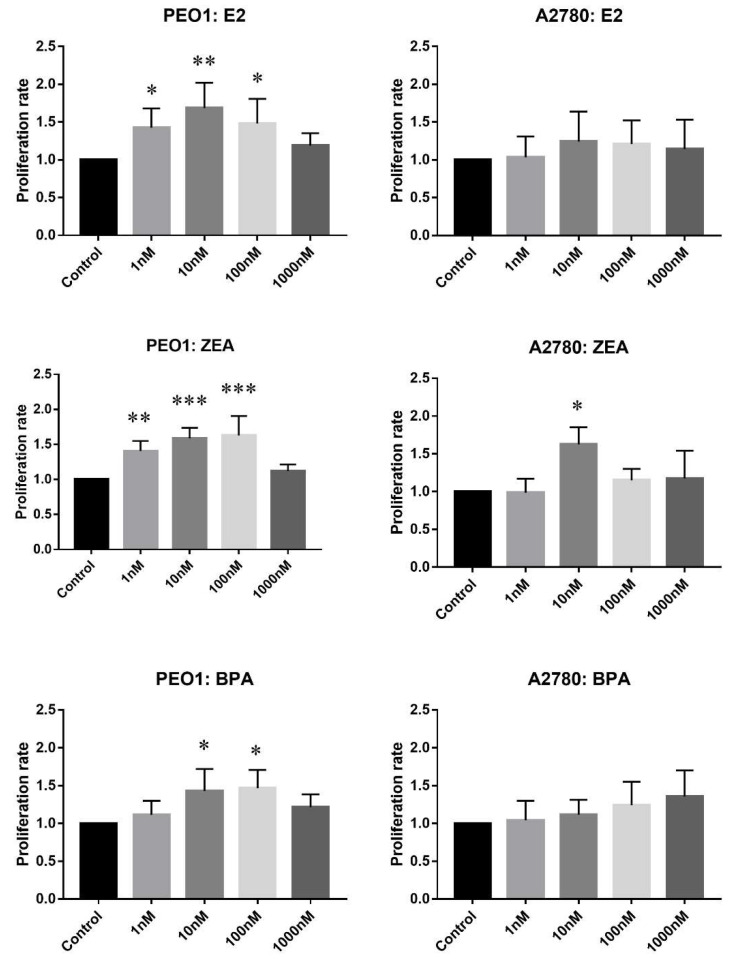
Effect of E2 (estradiol), ZEA (zeralaenone) and BPA (bisphenol A) on the proliferation of PEO1 and A2780 cell lines. Proliferation rate was determined by cell counting after the addition of 1, 10, 100, 1000 nM E2, ZEA and BPA. Cell count in case of the non-treated wells was regarded as 1. Data are presented as mean ± S.D. Significance was determined relative to the non-treated control: * *p* < 0.05; ** *p* < 0.01; *** *p* < 0.001.

In the proliferative assay, cell counts relative to the non-treated controls were compared. As Figure 1 shows, E2, ZEA and BPA increased the rate of cell proliferation in a concentration-dependent manner in the case of the PEO1 cell line. It is also important to mention that the effective doses of ZEA and E2 were highly comparable in contrast to the proliferative effect of BPA, which proved to be weaker (Figure 1). In the case of the A2780 cell line, only 10 nM ZEA had a small but significant effect on cell proliferation (Figure 1).

**Figure 2:**
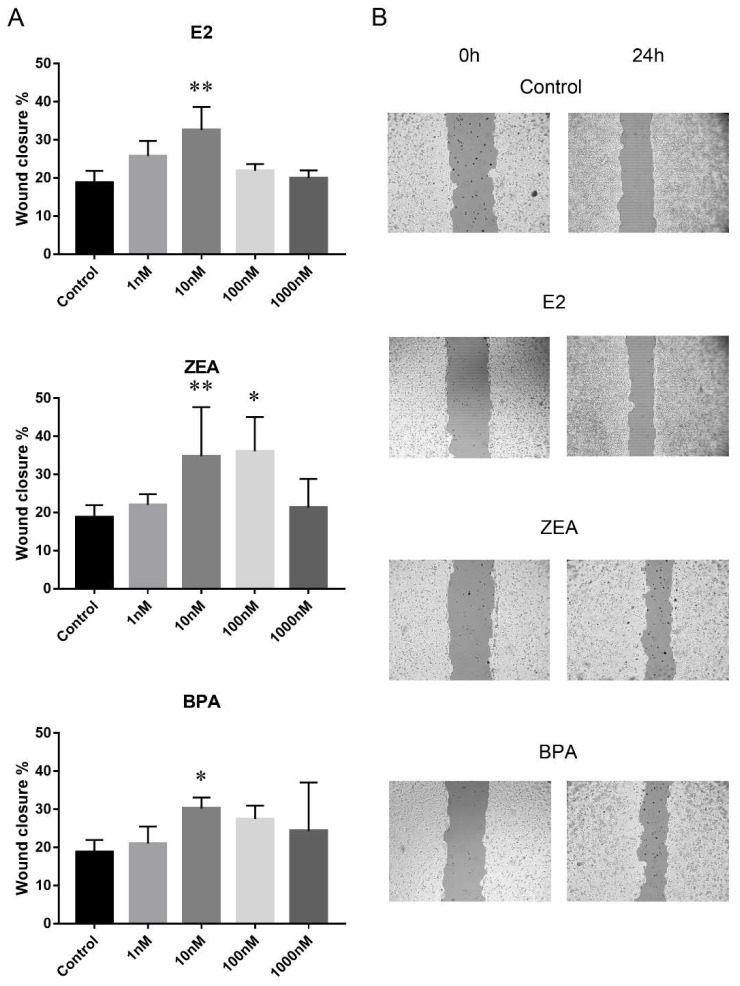
Effect of E2, ZEA and BPA on the migration of the PEO1 cell line. (**A**) Migration rate in response to the addition of 1, 10, 100, 1000 nM E2, ZEA and BPA. Migration rate was determined by scratch assay. Open wound area was determined at the time of scratching (0 h) and 24 h later. Wound closure % indicates migration rate. Data are presented as mean ± S.D. Significance was determined relative to the non-treated control: * *p* < 0.05; ** *p* < 0.01. (**B**) Representative result of a scratch assay experiment after the addition of 10 nM E2, ZEA and BPA. The output of the TScrach software is presented. Magnification: ×10.

Cell migration was studied by wound-healing scratch assay where wound closure % was used to indicate the rate of cell migration. The migration rate of the PEO1 and A2780 cell lines proved to be comparable 24 h after scratching in the non-treated control wells: wound closure % was 18.7 ± 3 in the case of the PEO1 cell line and 16.5 ± 2.6 in A2780. It is important to note that wound closure % was significantly higher in the A2780 cell line (36.1 ± 5) than in the PEO1 (24.3 ± 4) 48 h after scratching, which suggests a higher migrative ability of A2780. In order to compare the migrative effect of E2, ZEA and BPA molecules on the two cell lines, wound closure % was estimated 24 h after scratching because of the comparable migration rate of the two cell lines at this time point in the non-treated control wells. The addition of E2 and BPA increased the rate of migration in case of the PEO1 cell line at 10 nM concentration. ZEA proved to have the strongest migrative effect since migration was induced at both 10 and 100 nM doses (Figure 2). None of these molecules were able to further increase the rate of cell migration significantly in the A2780 cell line (Figure S1).

In order to characterize the toxic effect of these molecules to ovarian cells, apoptosis and cell lysis were studied. The decrease of mitochondrial membrane potential is an early marker of apoptosis that was determined by the DilC1(5) assay. According to our results, the rate of apoptosis was not comparable after the addition of E2, ZEA or BPA with the rate measured in case of the positive control in the tested cell lines (Figure S2). This suggests that the treatments did not induce apoptosis under our experimental circumstances. Cytotoxicity was also studied by the characterization of cell lysis where the activity of the cytosolic lactate dehydrogenase (LDH) enzyme was determined in the supernatant of the cultures. High LDH activity in the supernatant indicates cell membrane damage. No detectable LDH activity was found in the supernatant of the cultures of PEO1 or A2780 cell lines even after estrogen exposure (Figure S3). According to these results the estrogen treatments did not prove to be toxic to the tested cell lines at the concentration range used in our experiments.

### 2.2. Estradiol, Zearalenone and Bisphenol A Induce Comparable Changes in Gene Expression

The effect of E2, ZEA and BPA on gene expression was also compared. Based on previous data and biological significance, four estrogen-responsive genes were selected for gene expression analysis: *GREB1* is a highly estrogen-responsive growth regulator [28]; *DEPTOR* is an mTORC1 target [29]; *CA12* codes for an anhydrase [30]; *RBBP8* is involved in the regulation of DNA replication, transcription, in G1 and G2 checkpoint control and DNA repair [31]. All of these genes respond to estrogen exposure in breast cancer and their expression has been confirmed in ovarian cancer as well [32,33,34]. Because estrogen treatments proved to have migrative effect in the phenotypic studies, the expression of *CDH1* and *ZEB1* was also studied. *CDH1* codes for E-cadherin and its downregulation contributes to tumor cell invasion by promoting EMT of the tumor cells [35]. *ZEB1* is involved in the inhibition of *CDH1* expression and induces EMT [14].

**Figure 3:**
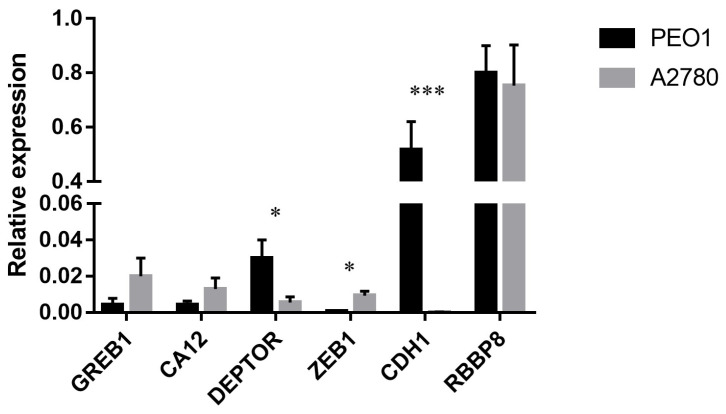
Relative expression level of *GREB1*, *CA12*, *DEPTOR*, *ZEB1*, *CDH1* and *RBBP8* in the PEO1 and A2780 cell lines. Relative expression was determined using *GAPDH* as reference. Total RNA was isolated from the cell lysates of non-treated cells. Data are presented as mean ± S.D. Significance was determined between the ΔCT values of the PEO1 and A2780 cell lines: * *p* < 0.05; *** *p* < 0.001.

The basal expression of *GREB1*, *CA12* and *RBBP8* was comparable in the estrogen-responsive PEO1 and non-responsive A2780 cell lines. However, the relative expression of *DEPTOR*, *CDH1* and *ZEB1* genes differed in the tested cell lines where the deviation in *CDH1* expression proved to be the most significant (Figure 3). The expression of these genes in response to estrogen treatments was also studied. In case of the PEO1 cell line *GREB1*, *DEPTOR*, *CA12* and *RBBP8* genes showed significant upregulation in response to estrogen treatments among which the upregulation of *GREB1* was the most remarkable (Table 1). According to the relative expression ratios, the highest response was induced by E2 compared to the non-treated control that was more comparable with ZEA than with BPA in the PEO1 cell line (Table 1). It is also important to mention that the *CDH1* gene was markedly under-expressed in response to the treatments (Table 1). In the case of the A2780 cell line, none of the tested molecules had a significant effect on the expression of *GREB1*, *DEPTOR*, *CA12*, *RBBP8* or *ZEB1* (Table 1).

### 2.3. Estradiol, Zearalenone and Bisphenol A Alter the Expression of MiR200s and MiR203a in a Time-Dependent Manner in ERα-Expressing Cells

**Figure 4:**
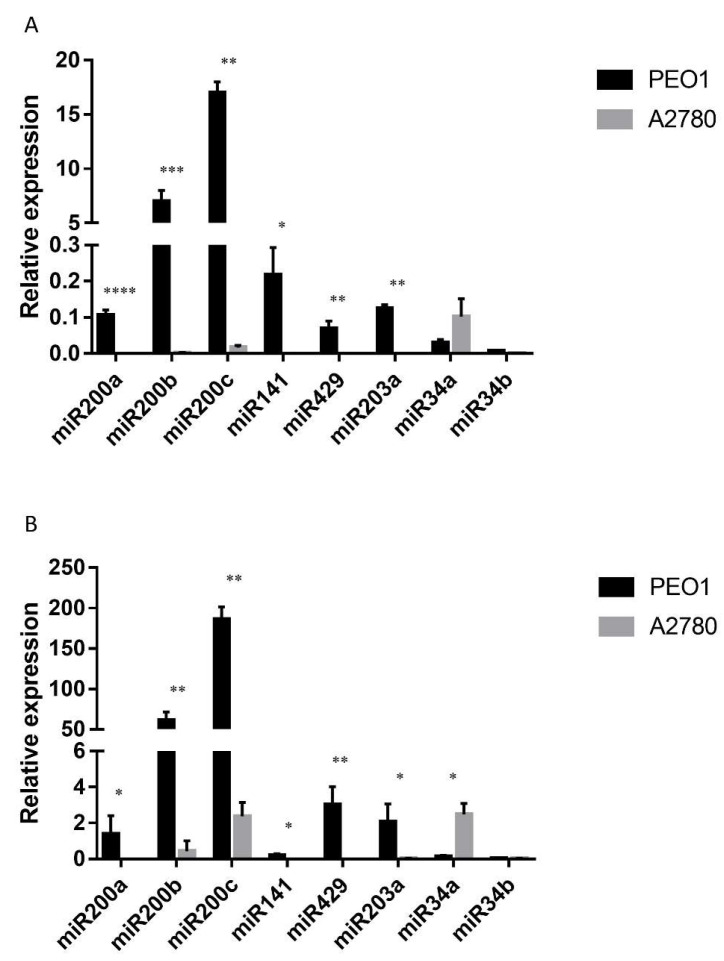
Relative expression level of miR200s, miR203a, miR34a and miR34b in the PEO1 and A2780 cell lines. Relative expression was determined using miR103 as reference. (**A**) Intracellular miRNA expression: MiRNA expression was detected in the cell lysates of non-treated cells. (**B**) Cell-free miRNA expression: MiRNAs were isolated from the supernatant of the cultures of non-treated cells. Data are presented as mean ± S.D. Significance was determined between the ΔCT values of the PEO1 and A2780 cell lines: * *p* < 0.05; ** *p* < 0.01; *** *p* < 0.001; **** *p* < 0.0001.

The expression level of miRNAs was also studied, since the miR200 family members (miR200a, miR200b, miR200c, miR141, miR429), miR34a, miR34b and miR203a are considered to play important roles in tumor cell migration by the regulation of *ZEB1* and *CDH1* [36,37,38]. Moreover, their cell-free counterparts proved to be promising biomarkers in the diagnosis of ovarian cancer according to our previous studies [24]. As Figure 4A shows, miR200s and miR203a were stably expressed in the estrogen-responsive PEO1 cell line among which miR200b and miR200c had the highest basal expression. However, the relative expression of miR34a and miR34b was hardly detectable (Figure 4A). On the contrary, the basal expression of miR200s and miR203a proved to be significantly lower in the ERα non-expressing A2780 cell line where miR34a showed the highest expression level (Figure 4A).

We also observed changes in miRNA expression in response to E2, ZEA and BPA exposure in the case of the PEO1 cell line (Table 2). Estrogen treatments altered the expression of miR200s and miR203a in a time-dependent manner (Table 2). MiR200a, miR200b, miR200c, miR141 and miR203a were significantly upregulated 12 h after the treatments compared to the non-treated control where the addition of E2 had the strongest effect (Table 2). On the contrary, the expression of miR200a, miR200b and miR200c was downregulated 24 h after estrogen exposure compared to the non-treated control (Table 2). In the case of the A2780 cell line, the otherwise extremely low expression of miR200s and miR203a was not increased significantly in response to estrogen treatments (Table S1). The expression of miR34a did not change significantly either (Table S1).

### 2.4. Cell-Free MiRNA Expression Correlates Well with Intracellular MiRNA Expression

The cell-free expression of miR200s, miR34a, miR34b and miR203a was also determined. According to our results, all the tested miRNAs were detectable in the supernatant of both cell lines, although the miRNA content of the supernatant was lower than what was observed in the cell lysates (ΔCT (supernatant-cell lysate) = 7.9 ± 1.9). A similar phenomenon was reported previously [39]. The lower extracellular level was also true for the expression of miR103, which was used as a reference miRNA for normalization. Due to this fact, the relative expression levels of the tested cell-free miRNAs proved to be higher than their intracellular counterparts (Figure 4B). It is important to note that the basal expressions of the cell-free miRNAs were highly comparable to what was measured intracellularly (Figure 4). The basal expression of miR200s was significantly higher in PEO1 than in the A2780 cell line (Figure 4B). It is also important to mention that miR200b and miR200c proved to have the highest cell-free expression level. The same phenomenon was observed in the A2780 cell line where the relative expression of miR34a proved to be the highest both intra- and extracellularly (Figure 4). In order to determine the correlation between the intracellular and cell-free miRNA levels, Pearson’s correlation coefficient was calculated for the measured levels. A high positive correlation was observed in both of the cell lines. In the PEO1 cell line, the correlation coefficient was r = 0.71 (*p* < 0.001), the same as in the A2780 cell line (r = 0.71 (*p* < 0.01)).

The effect of E2, ZEA and BPA treatments on the expression of cell-free miRNAs was also determined. In the case of the ZEA and BPA treatments, a similar tendency was observed in the medium as to what was observed intracellularly, i.e., a higher miRNA content was detected at 12 h which decreased 24 h after the treatment (Table 3). Notably, following E2 treatment the amounts of cell-free miR200b, miR200c and miR203a increased compared to the non-treated control (Table 3). In case of the A2780 cell line the expression rate of cell-free miR200s and miR203a was not influenced markedly by the E2, ZEA and BPA treatments, the same as that which was observed in the case of the intracellular miRNAs (Table S2).

**Figure 5:**
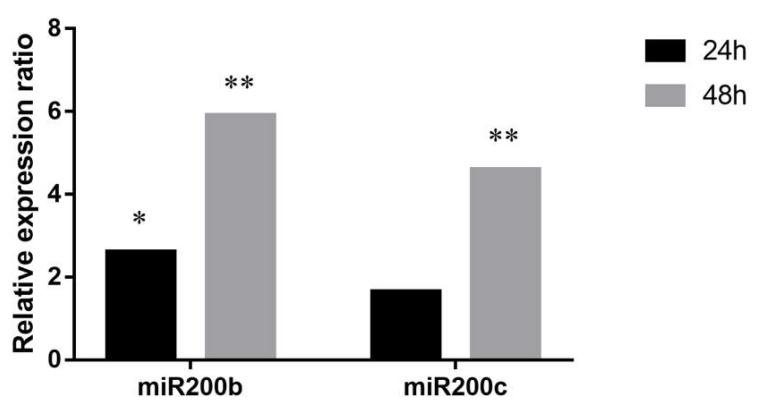
Relative expression ratios of miR200b and miR200c in A2780 cells co-cultured with PEO1. Relative expression ratio was calculated relative to the expression values of A2780 cultures without co-culturing (1). Ratio > 1 indicates upregulation relative to the non-co-cultured cell. Significance was determined between the ΔCT values of treated and non-treated samples: * *p* < 0.05; ** *p* < 0.01.

The high basal expression level of miR200b and miR200c suggests their high biological relevance in PEO1. We also investigated whether the cell-free counterparts of these miRNAs are able to influence the microenvironment through their transport into neighboring cells. In these experiments a co-culture assay was performed where PEO1 cells were seeded to a transwell chamber that was placed on the top of the A2780 cultures. Intracellular miR200b and miR200c levels of A2780 was determined by qPCR 24 and 48 h after co-culturing. According to our results the miR200b and miR200c levels of co-cultured A2780 cells increased significantly compared to the non-co-cultured cells in a time- dependent manner (Figure 5).

### 2.5. ERα is Involved in the Regulation of MiR200s and MiR203a Expression

**Figure 6:**
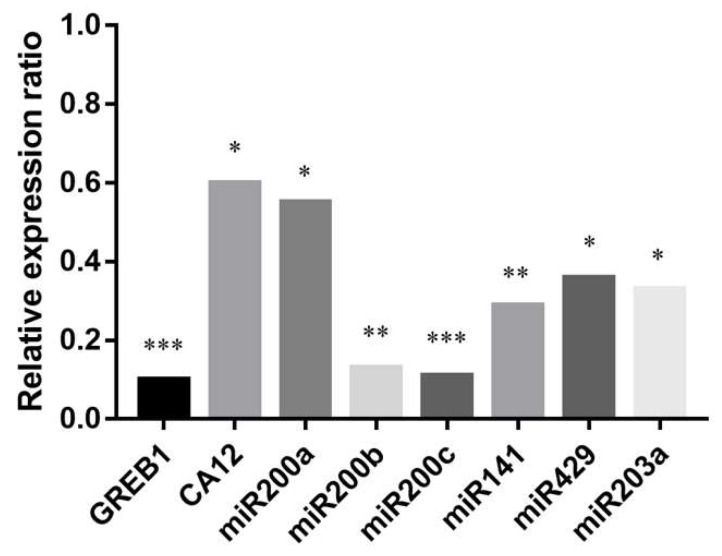
Relative expression ratios of *GREB1*, *CA12*, miR200s, and miR203a after the addition of ERα antagonist MPP (methyl-piperidino-pyrazole) along with E2. Relative expression was determined 12 h after the treatments. Relative expression ratio was calculated relative to the expression values of samples treated with E2 only (1). Ratio < 1 indicates downregulation relative to the E2-treated samples. Significance was determined between the ΔCT values of MPP treated and non-treated samples: * *p* < 0.05; ** *p* < 0.01; *** *p* < 0.001.

We aimed to investigate the ERα dependence of miR200s and miR203a expression. For this reason we added methyl-piperidino-pyrazole (MPP), a well-known ERα-selective antagonist [40], to the PEO1 cultures together with E2. Then, the expression of miR200s, miR203a and the highly estrogen-responsive *GREB1* and *CA12* genes was determined by qPCR. According to our results the effect of E2 was significantly decreased in the case of the miR200 family members and miR203a, as well as in the case of *GREB1* and *CA12*, after the addition of MPP (Figure 6).

**Figure 7:**
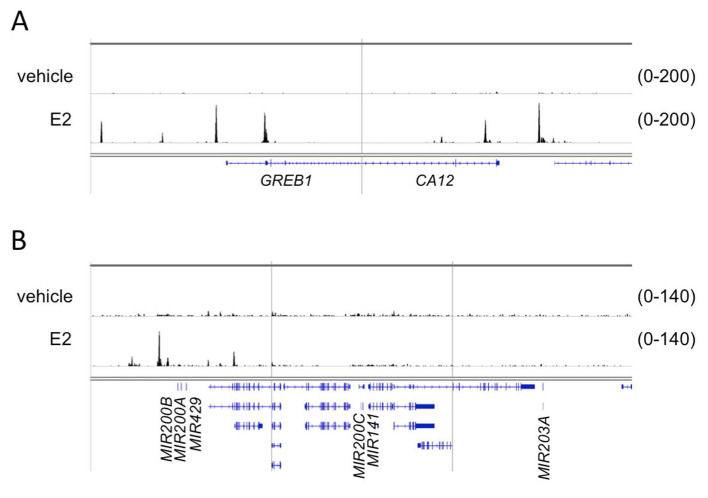
The genome browser view of ERα binding in case of the *GREB1*, *CA12*, miR200b-miR200a-miR429, miR200c-miR141 and miR203a genomic regions. ERα binding was determined in response to E2 treatment and in non-treated (vehicle) endometrial Ischikawa cells. (**A**) A 40 kb window around the transcription start site of two highly induced direct ER target genes, *GREB1* and *CA12*. (**B**) A 40 kb window around miRNA-encoding loci. For the sake of simplicity, gene symbols relevant to this study are indicated only on both panels.

In order to further strengthen our hypothesis we investigated whether the transcriptional induction of miR200s and miR203a could be associated by direct ERα binding at the cis-regulatory elements of these miRNAs coding loci. Since ERα is able to bind to promoter regions or distal enhancers, chromatin immunoprecipitation followed by sequencing (ChIP-seq), a method used to map protein–DNA interactions genome-wide, was applied to identify such putative cis-regulatory elements. We used publicly available ChIP-seq data for this purpose. At the time of our study, ERα ChIP-seq from human ovarian cells was not available, therefore, a ChIP-seq performed in endometrial Ischikawa cell line [41] was used for our analysis. The raw sequence files of control (vehicle) and cells stimulated with 10 nM E2 were downloaded from the database and analyzed by our ChIP-seq pipeline [42]. Five genomic loci were analyzed with respect to ERα binding (Figure 7). The first two regions covered *GREB1* and *CA12* protein coding genes, whose induction in response to E2 treatment was confirmed previously (Table 1). The additional three regions were (1) chr1:1147863–1187863, covering the *MIR200B-MIR200A-MIR429* locus (2) chr12:6943920–6983920, covering the *MIR200C-MIR141* locus and (3) chr14:104097437–104137437, covering *MIR203A* locus (Figure 7). The multiple binding sites of ERα associated with *GREB1* and *CA12* were in good agreement with the robust activation of these genes (Figure 7, Table 1). The analysis of the first genomic region covering *MIR200B-MIR200A-MIR429* revealed one high affinity binding site and few additional low affinity binding sites for ERα (Figure 7). We concluded that binding of ERα to these or putative other binding sites could contribute to transcriptional induction of these miRNAs. However, we could not identify ERα binding sites in the *MIR200C-MIR141* and *MIR203A* coding genomic loci (Figure 7).

## 3. Discussion

The role of estrogens in the development of breast cancer is a well-known phenomenon [3]. However, less information is available on their significance in ovarian cancer especially regarding xenoestrogens. In order to evaluate their role, we have conducted a comprehensive study in which the effect of ZEA and BPA was compared to E2, which is considered to be the most active form of natural estrogens and has been shown to induce an estrogen response in previous studies [2]. According to our results, E2, ZEA and BPA increased the rate of cell proliferation and migration which depended on the presence of ERα. Our results are in good agreement with previous studies where E2 proved to have proliferative and migrative effect on ERα-expressing cell lines, including PEO1, which was not observed in the absence of this receptor as in the case of A2780 [26,27,43,44]. The high overexpression of estrogen-responsive genes (*GREB1, CA12, DEPTOR, RBBP8*) after E2 exposure in PEO1 also supports the idea that the observed phenotype is due to gene expression changes induced by E2, which were not observable in A2780. ZEA and BPA were also able to induce cell proliferation and migration in several cell lines [45,46,47,48,49,50]. However, limited information is available on their effect on human ovarian cells. According to our studies, the effect of ZEA is highly comparable to that of E2 regarding the extent of the induced changes and the effective doses. The exposure to BPA also induced significant cell proliferation and migration, however, its effect seemed to be weaker. It is also worth mentioning that PEO1 responded well to these molecules at their physiologically relevant doses [51,52,53].

We also investigated whether the expression of miR200 and miR34 family members and miR203a is influenced by estrogens in human ovarian cells. These miRNAs are involved in the regulation of EMT according to previous studies, but they have an influence on other processes as well, including the cell cycle and angiogenesis [24,38,54,55]. According to our results, the basal expression of miR200 and miR34 family members as well as miR203a depended on ERα. MiR200s and miR203a showed high basal expression in the ERα-positive PEO1 cell line that correlated well with high *CDH1* and low *ZEB1* expression. This specific expression pattern is well explained by the fact that *ZEB1* is targeted by miR200s that promotes EMT by inhibiting E-cadherin expression [36]. A similar phenomenon was observed previously in several other cell lines [56]. Unexpectedly, the basal expression of miR34a, which is under the regulation of ERα in MCF7 cell lines [57], was higher in the absence of this receptor. Furthermore, the expression of miR200s and miR203a was altered in a time-dependent manner in response to E2, ZEA and BPA treatments. These miRNAs showed rapid upregulation to E2 treatment that was followed by their marked downregulation. Similar tendencies were observed in breast and endometrial cell lines [20,58,59]. It is important to mention that their decreased expression at the later time point is in good agreement with the downregulation of *CDH1* and enhanced migratory ability in response to estrogens in PEO1. The addition of ZEA and BPA also altered miRNA expression although these changes were not as remarkable as in the case of E2. Our results are in agreement with previous studies where BPA and ZEA altered miRNA expression in several other cell lines [20,60,61,62,63]. The observation that the effect of E2 on the expression of miR200s and miR203a was decreased by MPP, an ERα-selective antagonist, supports the presumption that ERα is involved in the regulation of these miRNAs. The role of ERα in the expression of miR200b, miR200a and miR429 is also supported by the analysis of publicly available ChIP-seq data of endometrial cells. However, no occupied ERα binding site was detected in the 40 kb window around the miR200c, miR141 and miR203a coding loci. This might be explained by the following reasons: (i) these miRNAs are regulated by ERα indirectly; (ii) ERα binds more distally from these miRNAs; (iii) ERα binding in this region is highly cell-type dependent. To address which is the most probable explanation, future ChIP-seq experiments performed in ovarian cells are required. The role of miR200b and miR200c in estrogen response was also suggested in breast cancer cells previously [64,65]. It was also described that the decrease of miR200a and miR200b expression in response to tamoxifen occurs via promoter inhibition of the *MIR200A-MIR200B-MIR429* locus [64,66]. However, to the best of our knowledge this is the first study that suggests the role of ERα in the transcriptional regulation of miR200s.

It is generally accepted that cell-free miRNAs might play prominent roles in cell-to-cell communication and influence the tumor microenvironment that favors tumor growth [22,23]. Our results suggest that cell-free miR200b and miR200c might be possible players of this process in ovarian cancer due to their supposed delivery to neighboring cells. It is interesting to mention that these miRNAs were able to affect the phenotype of several cell types in previous studies that favored tumor growth as well as cancer immune escape [67,68,69,70]. It is important to note that the presence of cell lysis or apoptosis was ruled out during the tested conditions, which suggests the active secretion of these miRNAs to the extracellular environment, a process which was also suggested by others [71]. The strong correlation between the intracellular and cell-free levels of miR200s and their ERα dependence suggest a promising future application of these cell-free miRNAs. Although ERα expression might play a prominent role in the development of ovarian tumors, the application of estrogen-blocking agents (such as tamoxifen) in the therapy of ovarian cancer has achieved mixed results [16,17]. This might be explained by the lack of available biomarkers for estrogen sensitivity of the tumor cells. According to our results, cell-free miR200s might be useful biomarkers for this purpose in non-invasive diagnostics. Among these, miR200b might be the best candidate due to the following reasons: (i) it showed a well-detectable expression level in ERα-expressing cells; (ii) its intracellular and cell-free expression level responded well to E2; (iii) its transcription is supposed to be regulated by ERα; (iv) its expression was well detectable in the plasma samples of patients with ovarian tumors and it proved to be an applicable biomarker in ovarian cancer in our previous studies [24]. These observations suggest that cell-free miR200s are not only promising biomarkers in the non-invasive diagnostics of ovarian cancer but might provide information about the pathophysiology of the tumor cells as well (e.g., ERα expression and estrogen responsiveness). This information might be useful in therapy selection (e.g., the application of estrogen disruptors) and support personalized medicine in the future.

## 4. Materials and Methods

### 4.1. Culturing Conditions

Human epithelial ovarian cell lines were used in the study. PEO1 was purchased from Merck (ECACC) and A2780 was kindly provided by Katalin Goda (University of Debrecen, Faculty of Medicine, Department of Biophysics and Cell Biology). Both of the cell lines were routinely cultured at 37 °C, 90% humidity and 5% CO_2_ in RPMI1640 (Corning, New York, USA) supplemented with 10% FBS (Corning, New York, SA), 1% l-glutamine, 100 µg/mL streptomycin and 100 U/mL penicillin. In order to study the effect of E2, ZEA and BPA, exponentially growing cells were harvested by trypsinization and plated to 96- or 24-well plates in RPMI1640 medium supplemented with 10% FBS. After attachment, the medium was removed and replaced with PRF-RPMI1640 (Corning, New York, USA) supplemented with 5% DCC-FBS (Corning, New York, USA). Cells were incubated for 24 h then E2, ZEA and BPA (Sigma-Aldrich, St. Louis, MO, USA) were added to the cells in different concentrations (1, 10, 100, 1000 nM). This time point was designated as 0 h. In some experiments, 1 nM MPP was also added to the cell cultures together with E2.

In order to determine the proliferative effect of the E2, ZEA and BPA treatments, cells were harvested when the non-treated control wells reached ~90% confluency (48 h after estrogen treatment in the case of A2780 and 72 h after the treatment in the case of PEO1) and were counted in a Bürker chamber. Relative growth rate was calculated from the results of 4 independent experiments where the cell count of treated wells was presented relative to the cell count of non-treated wells (1).

### 4.2. Migration Assay

Cell migration was determined by wound-healing scratch assay. Cells were plated to 24-well plates as previously described. After reaching 100% confluency a scratch was made with a pipette tip and the cells were treated with E2, ZEA and BPA. Three photos were taken from each well in the time of scratching (0 h) and 24 h later. Open wound area was determined by the TScratch software [72]. The rate of migration was determined from 4 independent experiments by wound closure % as described elsewhere [73]. In order to prevent the confounding effect of cell proliferation the experiments were also performed in the presence of cytosine β-D-arabinofuranoside hydrochloride (1 µM, Sigma-Aldrich, St. Louis, MO, USA), which is a selective inhibitor of DNA synthesis.

### 4.3. Apoptosis Assay

The decrease in mitochondrial membrane potential was used as a marker of apoptosis that was determined by the DilC1(5) Assay as described elsewhere [74]. Fluorescence signal was measured by a CLARIOstar Plus microplate reader (BMG Labtech, Ortenberg, Germany) at 630 nm excitation and 670 nm emission. Apoptosis was induced by the addition of CCCP (carbonyl cyanide m-chlorophenyl hydrazine, Thermo Fisher Scientific, Walthman, MA, USA) in the case of the positive control. Relative fluorescence was determined from 4 independent experiments where the fluorescence signal measured in the treated wells was presented relative to the fluorescence signal measured in the non-treated wells (1).

### 4.4. Determination of Cytotoxicity

The rate of cytotoxicity was determined by measuring LDH activity in the supernatant of cell cultures by the CyQUANT LDH Cytotoxicity Assay Kit (Thermo Fisher Scientific, Walthman, MA, USA) according to the manufacturer’s instructions. LDH activity was determined at 490 nm by a Multiskan sky microplate reader (Thermo Fisher Scientific, Walthman, MA, USA). Cell lysis was induced by the addition of lysis solution in the case of the positive control.

### 4.5. Co-Culture Assay

In order to determine the ability of cell-free miR200b and miR200c to influence the miRNA level of neighboring cells, a co-culture assay was performed [75]. PEO1 cells were seeded to Millicell cell culture inserts (0.4 µm pore size, Merck Millipore, Burlington, MA, USA) and incubated for 24 h in culturing medium. Then, the medium was replaced with PRF-RPMI1640 supplemented with 5% DCC-FBS and the cells were treated with 10 nM E2 in order to enhance the extracellular transfer of miR200s. Chambers containing PEO1 cells were placed in the 24-well plates where A2780 cells were plated previously. This time point was designated as 0 h. Total RNA including small RNAs was isolated from the A2780 cells after 24 and 48 h incubation. Relative expression ratio of co-cultured A2780 cells was determined relative to the relative expression of non-co-cultured A2780 cells as described in the Section 4.7.

### 4.6. MRNA Isolation and Quantification

Cells were plated to 24-well plates and treated with E2, ZEA and BPA molecules as previously described. The cells were treated in 10 nM concentration according to the phenotypic studies where both proliferation and migration were induced significantly at this concentration. Then, 24 h later, cells were harvested with a cell scraper and total RNA was isolated by RNeasy Mini Kit (Qiagen, Hilden, Germany) according to the manufacturer’s instructions.

The expression of *ESR1*, *GREB1*, *DEPTOR*, *CA12*, *RBBP8*, *CDH1* and *ZEB1* was detected by the QuantiTect SYBR Green RT-PCR Kit (Qiagen, Hilden, Germany). The PCR reaction mixture contained 100 ng total RNA. RNA was reverse transcribed at 50 °C for 30 min, then the reaction was denatured at 95 °C for 15 min, followed by 40 cycles of 94 °C for 15 s, 54 °C for 30 s and 72 °C for 30 s. Finally, a melting curve was generated by taking fluorescent measurements from 65 °C until 97 °C to ensure the detection of a single PCR product. All measurements were performed in triplicate. Relative expression was determined relative to *GAPDH* expression from 4 independent experiments (∆CT method). Relative expression ratios were determined in response estrogen treatments relative to the non-treated control samples (Ratio: relative expression level in treated samples/relative expression level in non-treated samples). Primer sequences are listed in Table 4.

### 4.7. Intracellular and Cell-Free MiRNA Isolation and Quantification

Cells were plated to 24-well plates and treated with E2, ZEA and BPA molecules in 10 nM concentration as previously described. Then, 12 and 24 h later cells were harvested with a cell scraper and total RNA including small RNAs was isolated by a miRNeasy Kit (Qiagen, Hilden, Germany) according to the manufacturer’s instructions. Cell-free miRNAs released from the cells were also extracted from the supernatant of the cultures as follows. The supernatant was centrifuged in these experiments (20 min, 20,000× *g*, 4 °C) in order to pellet detached cells and cell debris, then total RNA including small RNAs was extracted from a 200 µL sample with the miRNeasy Serum/Plasma Kit (Qiagen, Hilden, Germany) according to the manufacturer’s instructions. The concentration of extracted miRNA was determined by miRNA-specific fluorometric assay using a Qubit^®^ 2.0 Fluorometer (Thermo Fischer Scientific, Waltham, MA, USA).

The miScript PCR System (Qiagen, Hilden, Germany) was applied for the detection of mature miRNAs. Reverse transcription of mature miRNAs was performed by the miScript II RT Kit (Qiagen, Hilden, Germany). The expression of mature miR200a-3p, miR200b-3p, miR200c-3p, miR141-3p, miR429-3p, miR34a-5p, miR34b-3p, miR34c-3p and miR203a-3p was detected by Lightcycler 96 instrument (Roche, Pleasanton, CA, USA) with the miScript SYBR Green PCR Kit (Qiagen, Hilden, Germany) using miRNA specific probes (miScript primer assays; Qiagen, Hilden, Germany). The PCR reaction mixture contained 500 pg reverse transcription products. The reaction samples were first denatured at 95 °C for 15 min, followed by 50 cycles of 94 °C for 15 s, 55 °C for 30 s and 70 °C for 30 s. Finally, a melting curve was generated by taking fluorescent measurements from 40 °C until 85 °C to ensure the detection of a single PCR product. All measurements were performed in triplicate. MiR103-3p was used as a housekeeping miRNA that proved to be a reliable marker in the case of the plasma samples previously [24,76] and showed constant expression both in the intracellular and cell-free samples. Note that the expression of RNU6 was also monitored in the intracellular samples, in which the Ct value did not differ significantly from the Ct of miR103 (ΔCT (CT RNU6-CT miR103) = 0.3 ± 0.1). However, in case of the cell-free miRNAs the expression of RNU6 was hardly detectable in some cases. Because of this reason, miR103 was used as a reference miRNA in all calculations. Relative expression level was determined relative to miR103 expression from 4 independent experiments (∆CT method). Relative expression ratios were determined in response to estrogen treatments relative to the non-treated control samples (ratio: relative expression level in treated samples/relative expression level in non-treated samples). Note that the presence of the studied miRNAs was not detectable in the cell-free culturing medium (data not shown).

### 4.8. Analysis of Chip-Seq Data

The following ChIP-seq fastq files were obtained from the SRA database: SRR6653434 (vehicle control) and SRR6653432 (E2-stimulated) [41]. The primary analysis of raw ChIP-seq reads was carried out using our ChIP-seq analysis pipeline installed on the local HPC cluster (Genomic Medicine and Bioinformatics Core Facility, Department of Biochemistry and Molecular Biology, Faculty of Medicine, University of Debrecen) [42]. Briefly, the Burrows-Wheeler Alignment Tool [77] was used to align the reads with the GRCh38 (hg38) human genome assembly, and Model-based Analysis of ChIP-Seq 2 (MACS2), [78] was used for predicting peaks. Bedtools was used to generate Genome coverage files (BedGraphs) from BAM files, and BedGraphs files were converted into tdf files using igvtools with the “toTDF” option. Integrative Genomics Viewer (IGV, Broad Institute) was used for creating representative snapshots [79].

### 4.9. Statistical Analysis

Statistical significance was studied by Student’s *t*-test. Multiple analysis was performed by one-way ANOVA with Dunnet’s test as post hoc analysis. Pearson’s correlation was used to determine the correlation between the expression values of miRNAs in the cell lysates and supernatant of non-treated samples. In these calculations the expression values of miRNAs were grouped in the non-treated samples and correlation was determined between the ΔCT values of intracellular and cell-free miRNAs. Statistical analysis and figures were made by GraphPad Prism 7.

## 5. Conclusions

Cell-free miRNAs represent a highly promising but understudied area of miRNA biology which might open new avenues in cancer research. Accumulating evidence suggests that cell-free miRNA expression correlates with the occurrence of several malignancies, and thus they are considered to be promising biomarker candidates in liquid biopsy [21,80]. However, less research is available on their biological significance or their application as biomarkers in cell cultures. Here we present that the expression of cell-free miRNAs correlates well with their intracellular counterparts, which suggests a much broader application of cell-free miRNAs: (i) they might be used as extracellular markers in cellular studies to monitor cellular physiology and/or target mRNA expression; (ii) they may be used in the development of cellular assays in biotechnology; (iii) their application in liquid biopsy as diagnostic biomarkers might be extended to providing information about the pathophysiology of tumor cells (e.g., ERα expression and estrogen/tamoxifen sensitivity).

## Figures and Tables

**Table 1:** Relative expression ratio of *GREB1*, *DEPTOR*, *CA12*, *RBBP8* and *CDH1* in response to estrogen treatments in the PEO1 and A2780 cell lines. Total RNA was isolated from the cells 24 h after E2, ZEA and BPA treatments. Relative expression ratio was calculated relative to the expression value of their respective mRNAs in the non-treated control (1). Notes: Ratio > 1 indicates upregulation relative to the non-treated control. Ratio < 1 indicates downregulation relative to the non-treated control. Significance was determined between the ΔCT values of treated and non-treated samples: * *p* < 0.05; ** *p* < 0.01; *** *p* < 0.001. n.d.-not detectable.

	E2		ZEA		BPA	
	PEO1	A2780	PEO1	A2780	PEO1	A2780
*GREB1*	263.19 ***	0.76	233.94 ***	0.79	20.53 **	0.58
*DEPTOR*	5.39 *	0.97	15.03 *	0.84	3.03 *	0.52
*CA12*	37.01 **	0.72	13.45 *	0.61	2.79 *	0.69
*RBBP8*	9.58 *	0.71	5.21 *	0.81	2.13 *	0.76
*CDH1*	0.42 *	n.d.	0.18 *	n.d.	0.44 *	n.d.
*ZEB1*	n.d.	1.18	n.d.	1.01	n.d.	0.75

**Table 2:** Relative expression ratios of miR200s, miR34s and miR203a after estrogen treatments in the PEO1 cell line. MiRNA was isolated from the cells 12 and 24 h after E2, ZEA and BPA exposure. Relative expression ratio was calculated relative to the expression value of miRNAs in the non-treated control (1). Ratio > 1 indicates upregulation relative to the non-treated control. Ratio < 1 indicates downregulation relative to the non-treated control. Significance was determined between the ΔCT values of treated and non-treated samples: * *p* < 0.05.

		miR200a	miR200b	miR200c	miR141	miR429	miR203a
E2	12 h	1.52 *	2.9 *	3.28 *	3.32 *	1.14	1.73 *
	24 h	0.56	0.28 *	0.43 *	0.95	1.09	0.82
ZEA	12 h	1.29	0.99	1.71 *	1.16	0.97	1.27
	24 h	0.22 *	0.42 *	0.74	0.71	0.64	0.68
BPA	12 h	1.89 *	2.91 *	2.12 *	1.75 *	1.85 *	1.27
	24 h	0.22 *	0.67	1.34	1.29	1.47	0.95

**Table 3:** Relative expression ratios of cell-free miR200s and miR203a after estrogen exposure in the PEO1 cell line. MiRNA was isolated from the supernatant of cell cultures 12 and 24 h after E2, ZEA and BPA treatments. Relative expression ratio was calculated relative to the expression value of miRNAs in the non-treated control (1). Ratio > 1 indicates upregulation relative to the non-treated control. Ratio < 1 indicates downregulation relative to the non-treated control. Significance was determined between the ΔCT values of treated and non-treated samples: * *p* < 0.05.

		miR200a	miR200b	miR200c	miR429	miR203a
E2	12 h	0.058 *	1.95 *	1.38	1.51	0.49 *
	24 h	0.042 *	1.57 *	1.57 *	1.01	1.8 *
ZEA	12 h	3.29 *	1.59 *	1.16	1.18	1.19
	24 h	0.08 *	0.38 *	0.34 *	0.44 *	0.56
BPA	12 h	1.89 *	1.71 *	1.94 *	1.19	1.64 *
	24 h	0.09 *	0.36 *	0.68	0.69	1.06

**Table 4:** Primer sequences for the RT-PCR analysis of mRNAs.

	Forward	Reverse
*ESR1*	GTGACTTCAATGGCGAAGG	TTCCCTTGTCATTGGTACTGG
*GREB1*	TCTTGCACAATTCCATCGAG	GTCCACTCGGCTACCACCT
*DEPTOR*	TCATGGCATCTGAATTCCTG	ATGGGTGCTTGTTGGACAC
*CA12*	GTTTCTCCTGACCAACAATGG	CGTGGCACTGTAGCGAGAC
*RBBP8*	CACTCAGACTTGTATGGAAAGAGG	TCCTTCTGTTTCTGTTTCAACG
*CDH1*	TCTGTGAGAGGAATCCAAAGC	TGGGAGGATCACTAGGTTCAA
*ZEB1*	AGGATGACCTGCCAACAGAC	ATTTCTTGCCCTTCCTTTCC
*GAPDH*	CACCCACTCCTCCACCTTT	GCCAAATTCGTTGTCATACCA

## Supplementary Materials

The following are available online at https://www.mdpi.com/1422-0067/21/24/9725/s1. **Figure S1.** Effect of E2, ZEA and BPA on the migration of the A2780 cell line. Migration rate was determined by Scrach assay after the addition of 1, 10, 100, 1000 nM E2, ZEA and BPA. Open wound area was determined at the time of scratching and 24 h later. Wound closure % indicates migration rate. Data are presented as mean ± S.D. **Figure S2.** Relative fluorescence in response to E2, ZEA and BPA treatments in the PEO1 and A2780 cell lines. Fluorescence signal measured in the non-treated wells was regarded as 1. In the case of the positive control apoptosis was induced by CCCP. Data are presented as mean ± S.D. **Figure S3.** Cytotoxicity in response to E2, ZEA and BPA treatments. Cell lysis was determined by LDH activity measured in the supernatant of the cells. In the case of the positive control cell lysis was induced by lysis buffer. Data are presented as mean ± S.D. **Table S1.** Relative expression of miR200s, miR34s and miR203a after estrogen treatments in the A2780 cell line. MiRNA was isolated from the cells 12 and 24 h after E2, ZEA and BPA treatments. **Table S2.** Relative expression of cell-free miR200s, miR34s and miR203a after estrogen treatments in the A2780 cell line. MiRNA was isolated from the cells 12 and 24 h after E2, ZEA and BPA treatments.

## Abbreviations

E2	Estradiol
ZEA	Zearalenone
BPA	Bisphenol A
ER	Estrogen receptor
EMT	Epithelial to mesenchymal transition
MiRNA	microRNA
MPP	methyl-piperidino-pyrazole
Chip-seq	Chrompatin immunoprecipitation followed by sequencing
