# Supplementary material for: The Cell-Free Expression of MiR200 Family Members Correlates with Estrogen Sensitivity in Human Epithelial Ovarian Cells
Source: Int J Mol Sci. 2020 Dec 20;21(24):9725. doi: 10.3390/ijms21249725 (PMC7766742; doi:10.3390/ijms21249725)
Supplement: Supplementary file 1 [file ijms-21-09725-s001.pdf]

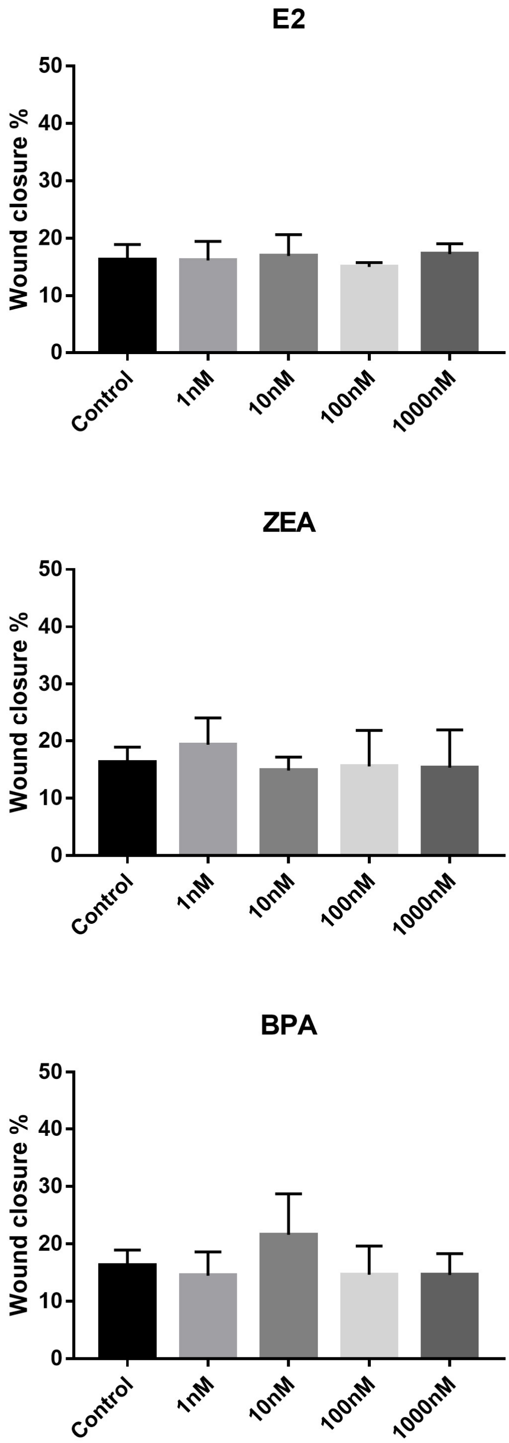

Supplementary Figure 1. Effect of E2, ZEA and BPA on the migration of the A2780 cell line. Migration rate was determined by Scrach assay after the addition of 1, 10, 100, 1000 nM E2, ZEA and BPA. Open wound area was determined at the time of scratching and 24 h later. Wound closure % indicates migration rate. Data are presented as mean ± S.D.

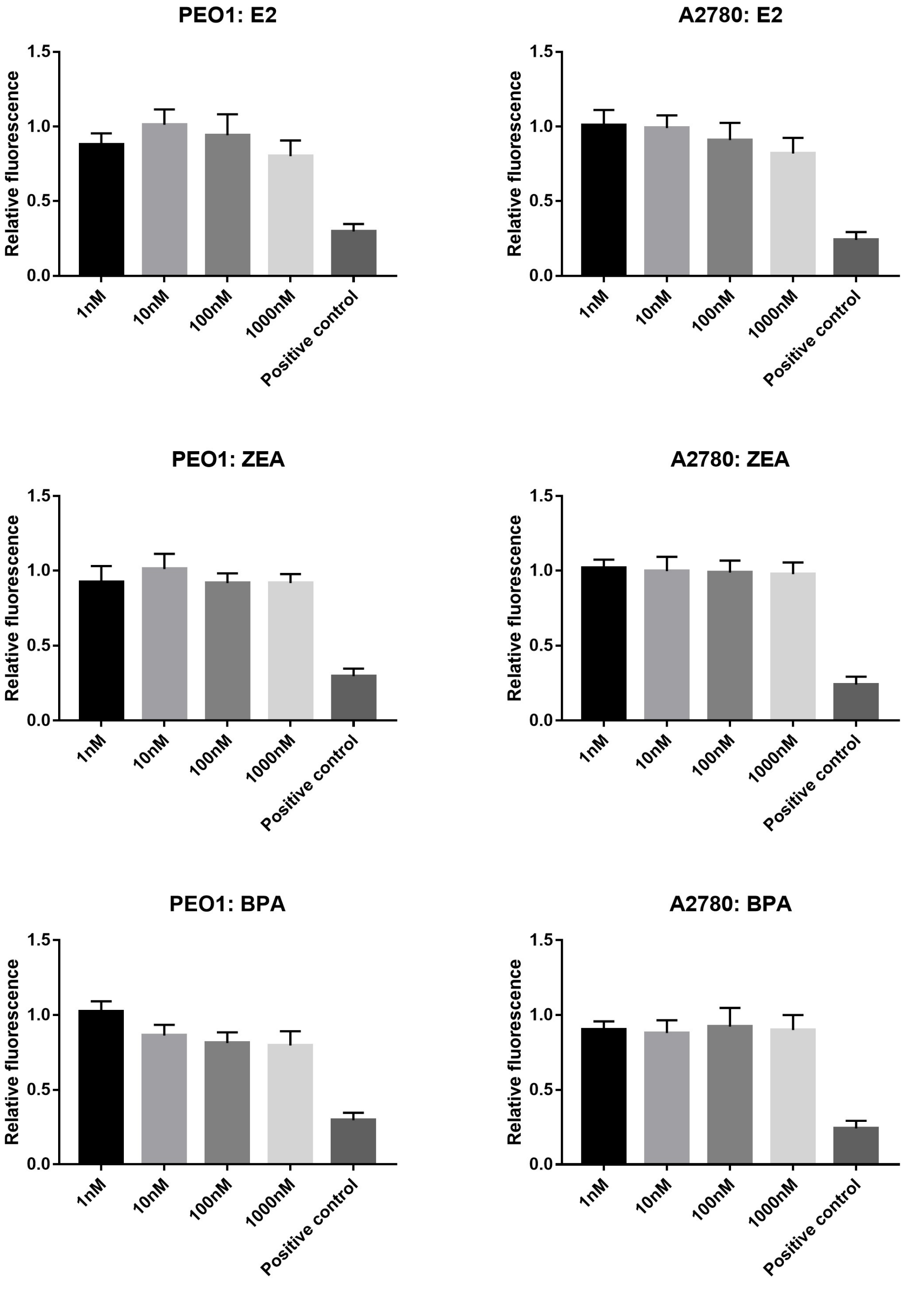

Supplementary Figure 2. Relative fluorescence in response to E2, ZEA and BPA treatments in the PEO1 and A2780 cell lines. Fluorescence signal measured in the non-treated wells was regarded as 1. In the case of the positive control apoptosis was induced by CCCP. Data are presented as mean ± S.D.

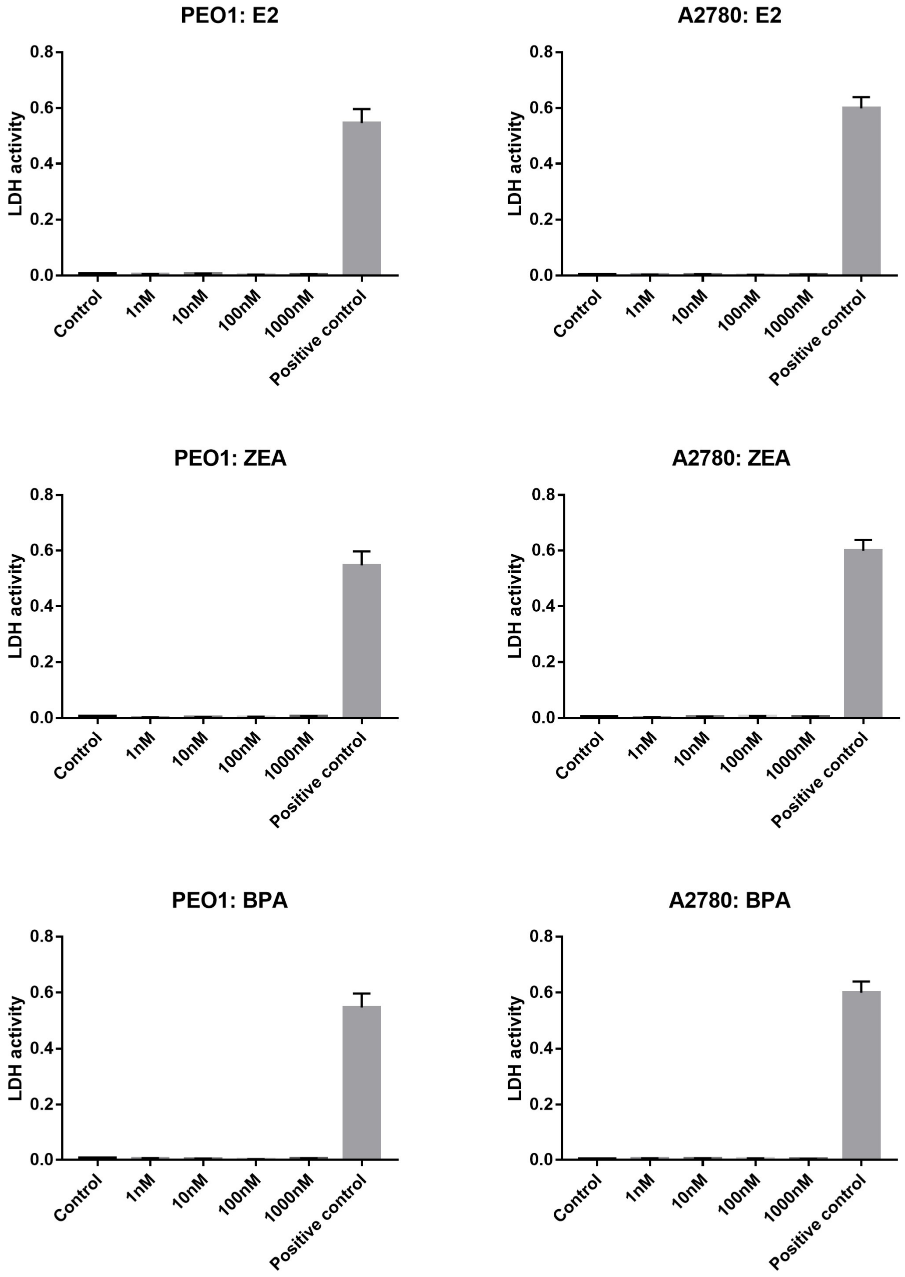

Supplementary Figure 3. Cytotoxicity in response to E2, ZEA and BPA treatments. Cell lysis was determined by LDH activity measured in the supernatant of the cells. In the case of the positive control cell lysis was induced by lysis buffer. Data are presented as mean ± S.D.

Supplemetary Table 1. Relative expression of miR200s, miR34s and miR203a after estrogen treatments in the A2780 cell line. MiRNA was isolated from the cells 12 and 24 h after E2, ZEA and BPA treatments.

		miR200a	miR200b	miR200c	miR141	miR429	miR34a	miR34b	miR203a
**Control**	**12h**	0	2E-03	2.1E-02	9.6E-04	3.3E-04	8E-02	5.7E-04	1E-05
	**24h**	1E-04	2E-03	1.7E-02	5.6E-04	2.2E-04	1.1E-01	7.6E-04	1E-05
**E2**	**12h**	9.5E-09	2.3E-03	1.7E-02	6.7E-04	4.1E-04	9E-02	6.5E-04	8.1E-05
	**24h**	9.1E-06	2.2E-03	2.4E-02	1.2E-03	4.7E-04	1.1E-01	7.1E-04	2.7E-05
**ZEA**	**12h**	2.1E-05	1.2E-04	1.9E-02	2.9U-04	2.7E-04	7E-02	5.1E-04	7.7E-06
	**24h**	7.4E-05	3.3E-03	2.4E-02	7.3E-06	3.E-05	1.1E-01	8.4E-04	1.4E-05
**BPA**	**12h**	1.3E-08	1.51E-06	2.7E-02	1.1E-03	2.2E-04	1.2E-01	1.5E-04	1.2E-06
	**24h**	3.7E-07	2.4E-03	2.4E-02	8.9E-04	1.7E-04	8E-02	7.8E-04	2E-06

Supplemetary Table 2: Relative expression of cell free miR200s, miR34s and miR203a after estrogen treatments in the A2780 cell line. MiRNA was isolated from the cells 12 and 24 h after E2, ZEA and BPA treatments.

		miR200a	miR200b	miR200c	miR141	miR429	miR34a	miR34b	miR203a
**Control**	**12h**	4.4E-04	8.4E-02	3.3E-01	2E-05	7E-05	2.3E-01	2.3E-02	1.1E-01
	**24h**	3.4E-04	9.2E-02	2.6	1E-05	2.8E-04	2.6	1.9E-02	2.3E-02
**E2**	**12h**	3.3E-04	1.5E-01	1.3E-01	1.1E-04	1.1E-02	5.4E-03	2.9E-02	3.3E-01
	**24h**	2.5E-04	1.5E-01	2.2E-01	5.5E-04	2.8E-05	4.3E-03	1.5E-03	1.6E-01
**ZEA**	**12h**	4.7E-04	1.5E-03	2.5E-01	1.3E-05	1.5E-05	5.8E-01	7.5E-04	2.1E-01
	**24h**	3.4E-04	6.5E-02	2.5E-01	1.6E-03	6.2E-05	8.1E-01	5.9E-04	7.2E-02
**BPA**	**12h**	2.4E-04	8.2E-02	2.9E-01	3.2E-04	1.9E-03	2.8E-01	1.9E-02	1.9E-01
	**24h**	5.6E-04	1.9E-01	4.7E-01	4.7E-04	3.5E-04	1.3E-01	9E-03	2.5E-02
